# Supplementary material for: Aurora B Kinase Inhibition by AZD1152 Concomitant with Tumor Treating Fields Is Effective in the Treatment of Cultures from Primary and Recurrent Glioblastomas
Source: Int J Mol Sci. 2023 Mar 6;24(5):5016. doi: 10.3390/ijms24055016 (PMC10003311; doi:10.3390/ijms24055016)
Supplement: Supplementary file 1 [file ijms-24-05016-s001.zip › ijms-2131865-supplementary.pdf]

Figure S1

Cytotoxic effect in established cell lines

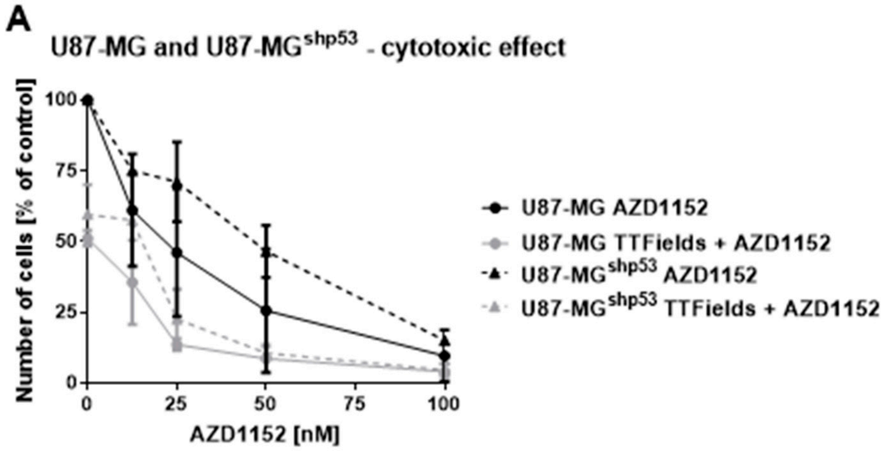

Figure S1: Presenting data from Figure 1 in one graph for direct comparison. Cytotoxic effect in established cell lines. Increased efficacy of the concomitant treatment of AZD1152 and TTFields in U87-MG and U87-MG^{shp53} cells. Both glioma cell lines were treated with various AZD1152 concentrations and TTFields (200 kHz, 1.6 V/cm RMS) for 72 hours. (**A**) The number of cells was determined at the end of treatment and is expressed as percentage of control. Data represent mean ± SD (N = 2).

Figure S2

Enlarged (x10) views from Figure 1B

U87-MG 0 nM AZD1152

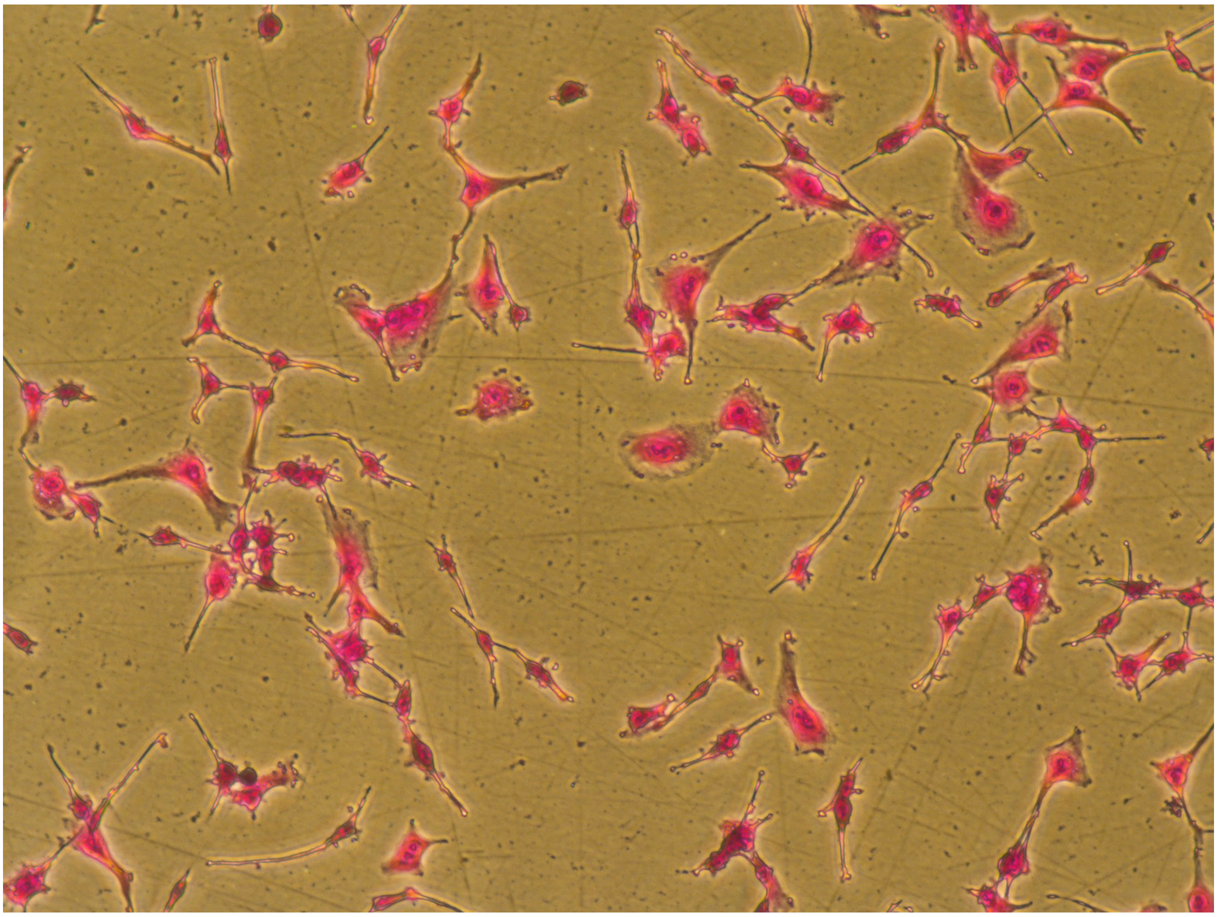

U87-MG 0 nM AZD1152 + TTFields

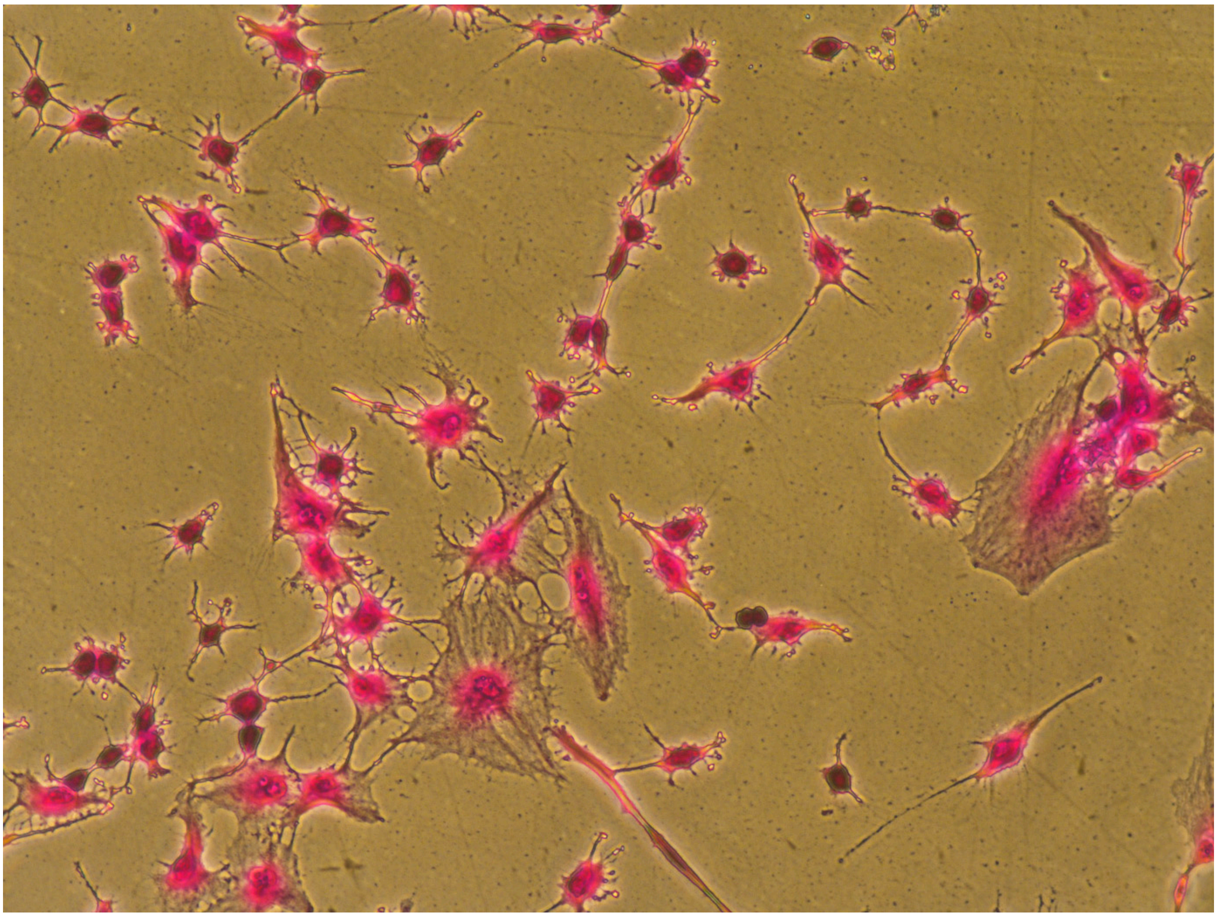

U87-MG 25 nM AZD1152

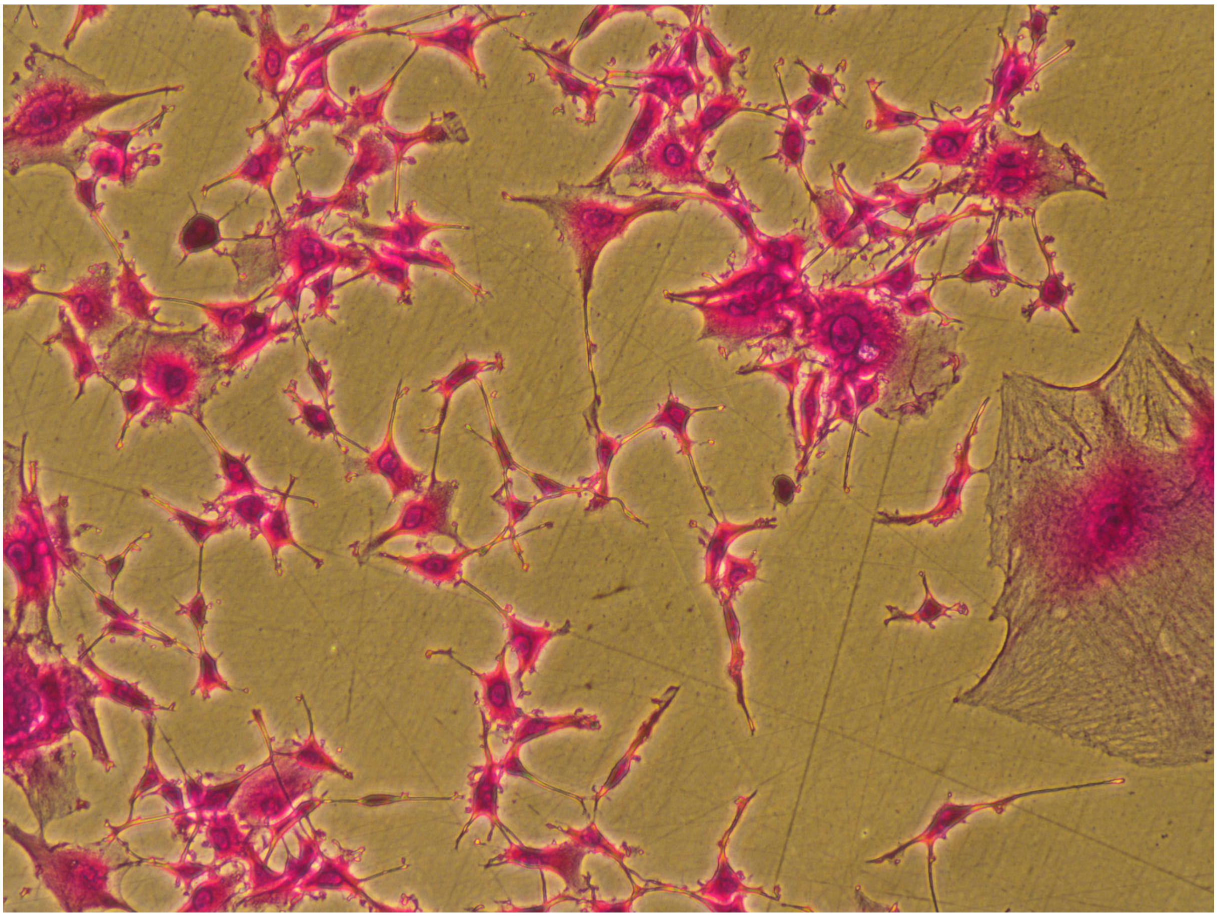

U87-MG 25 nM AZD1152 + TTFields

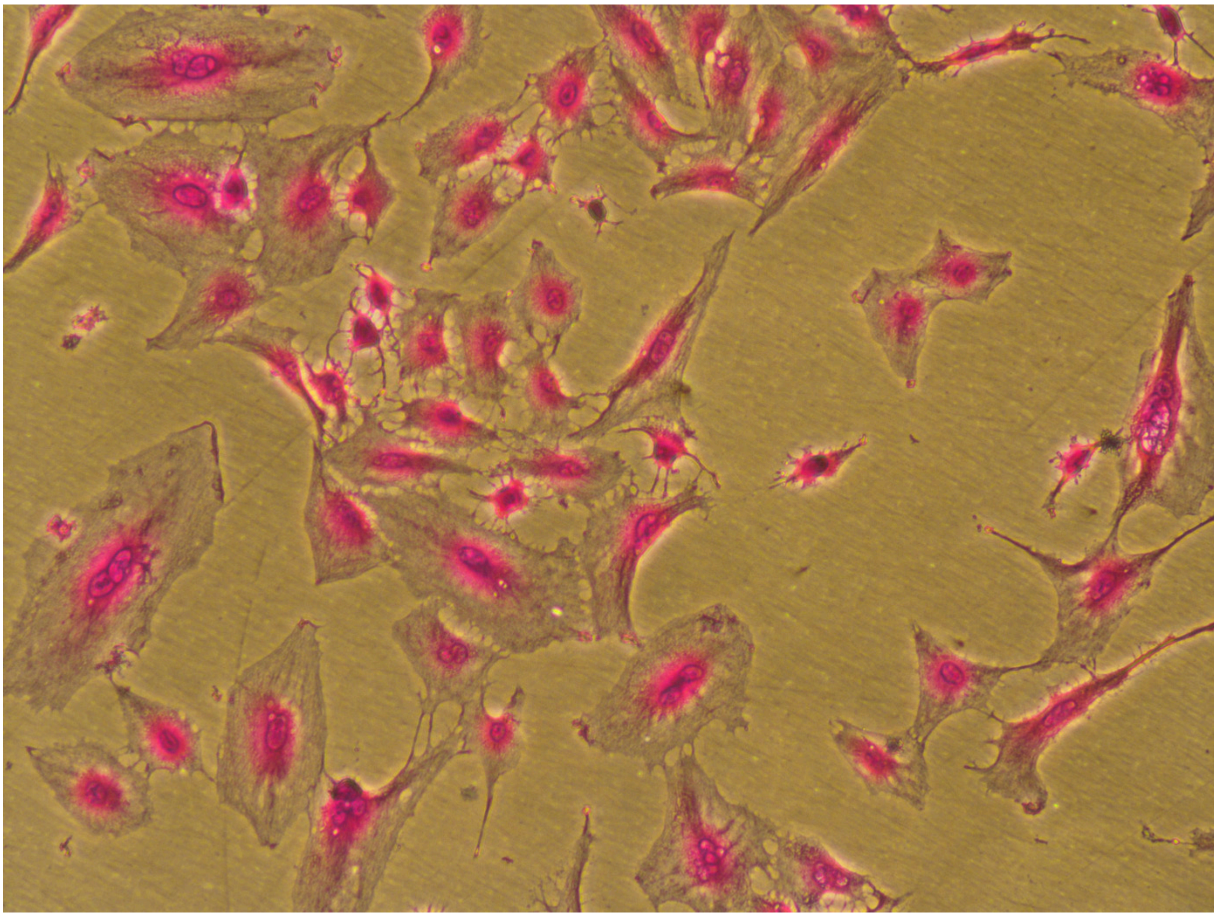

U87-MG 50 nM AZD1152

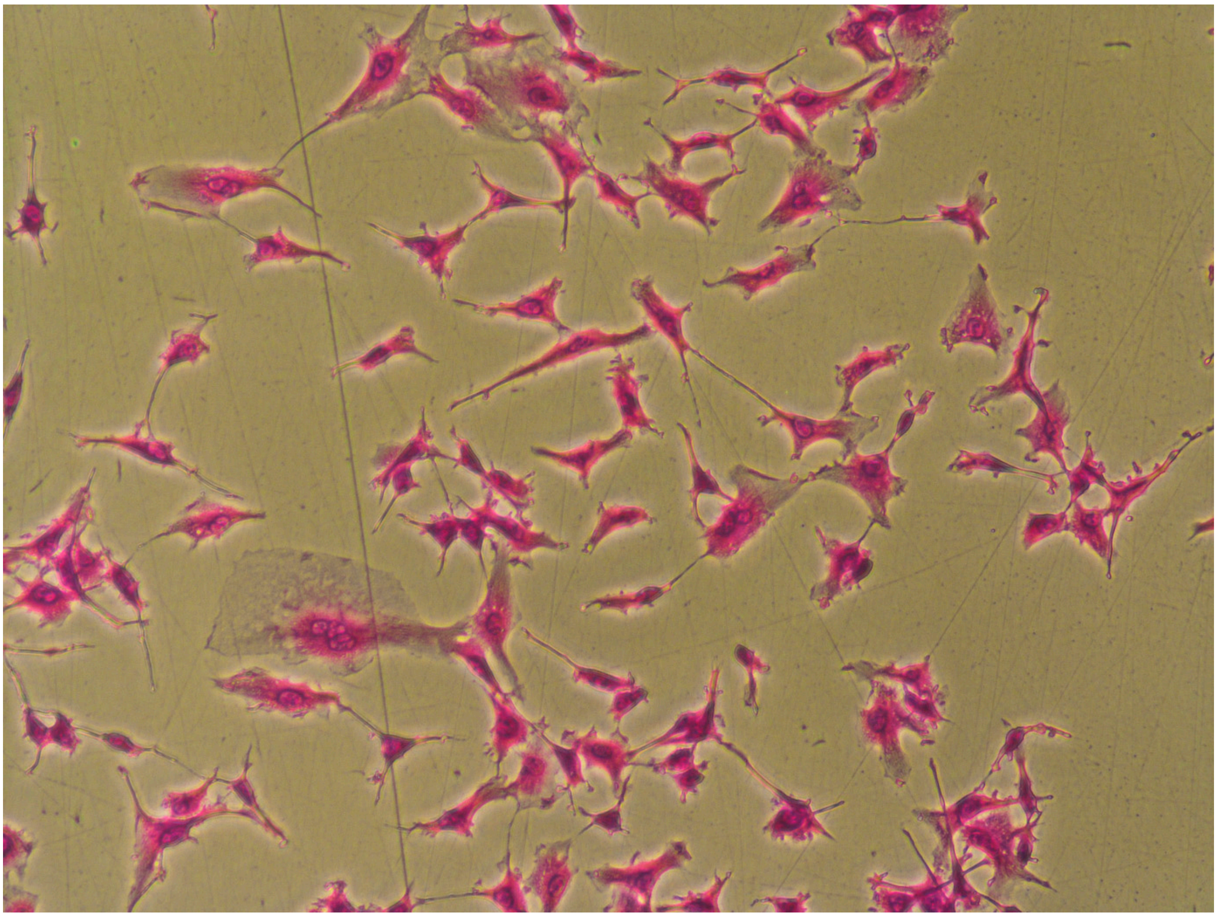

U87-MG 50 nM AZD1152 + TTFields

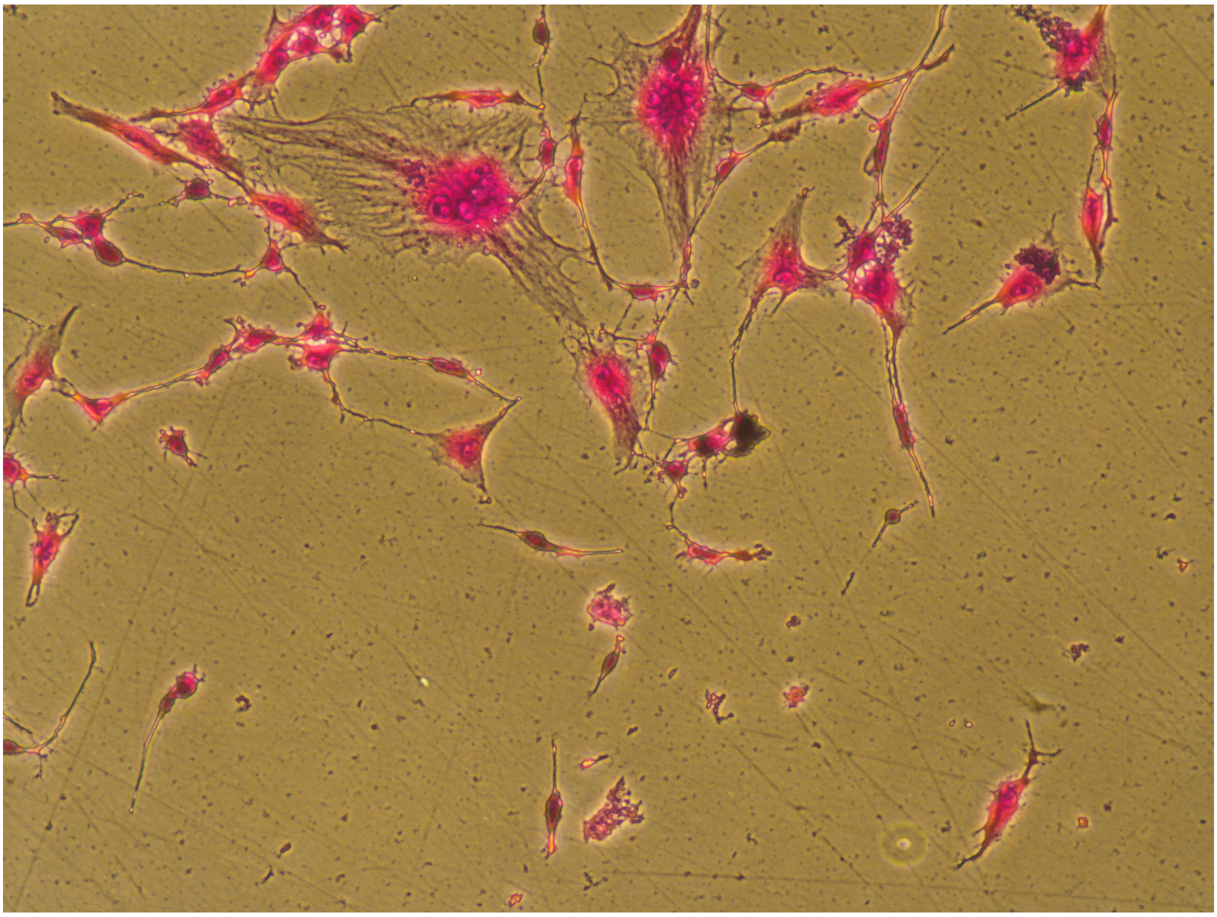

U87-MG 100 nM AZD1152

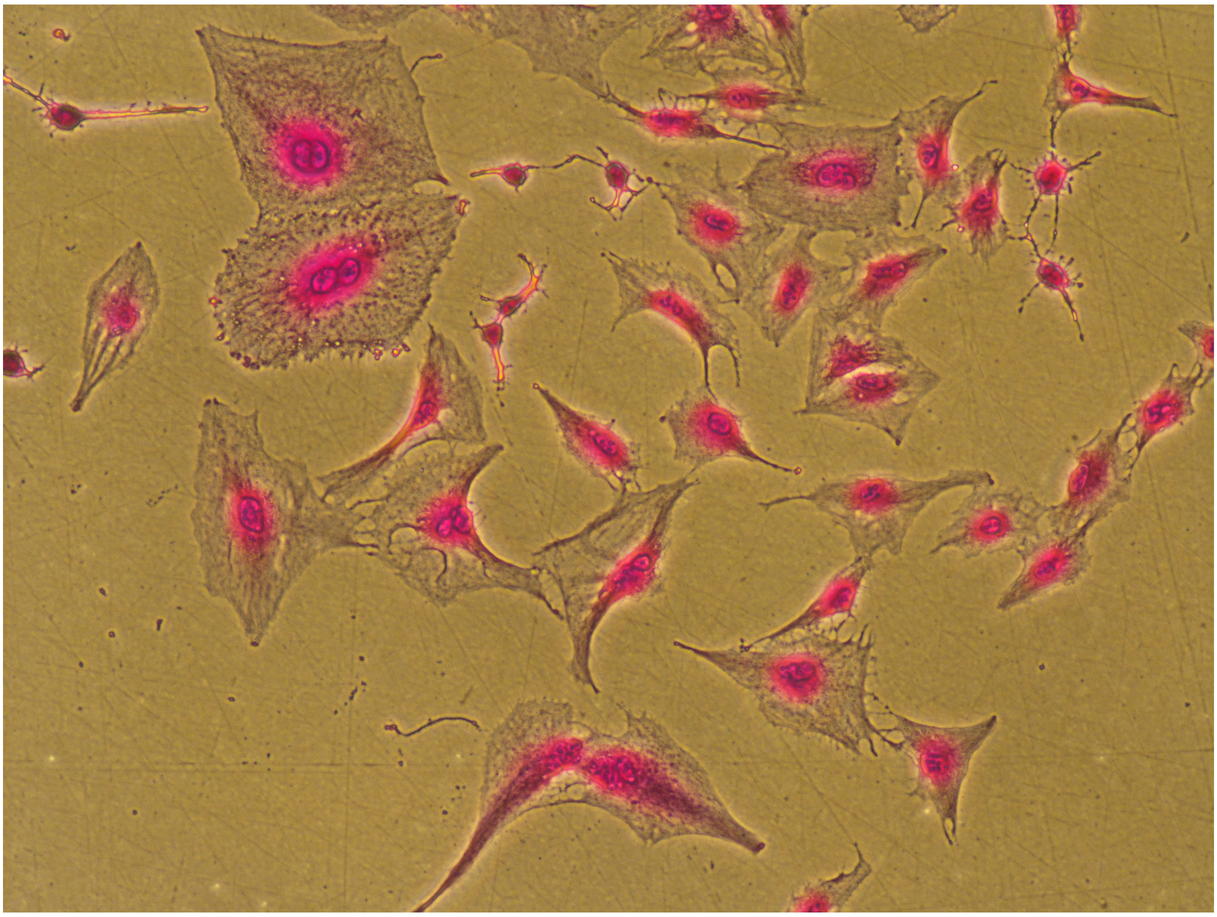

U87-MG 100 nM AZD1152 + TTFields

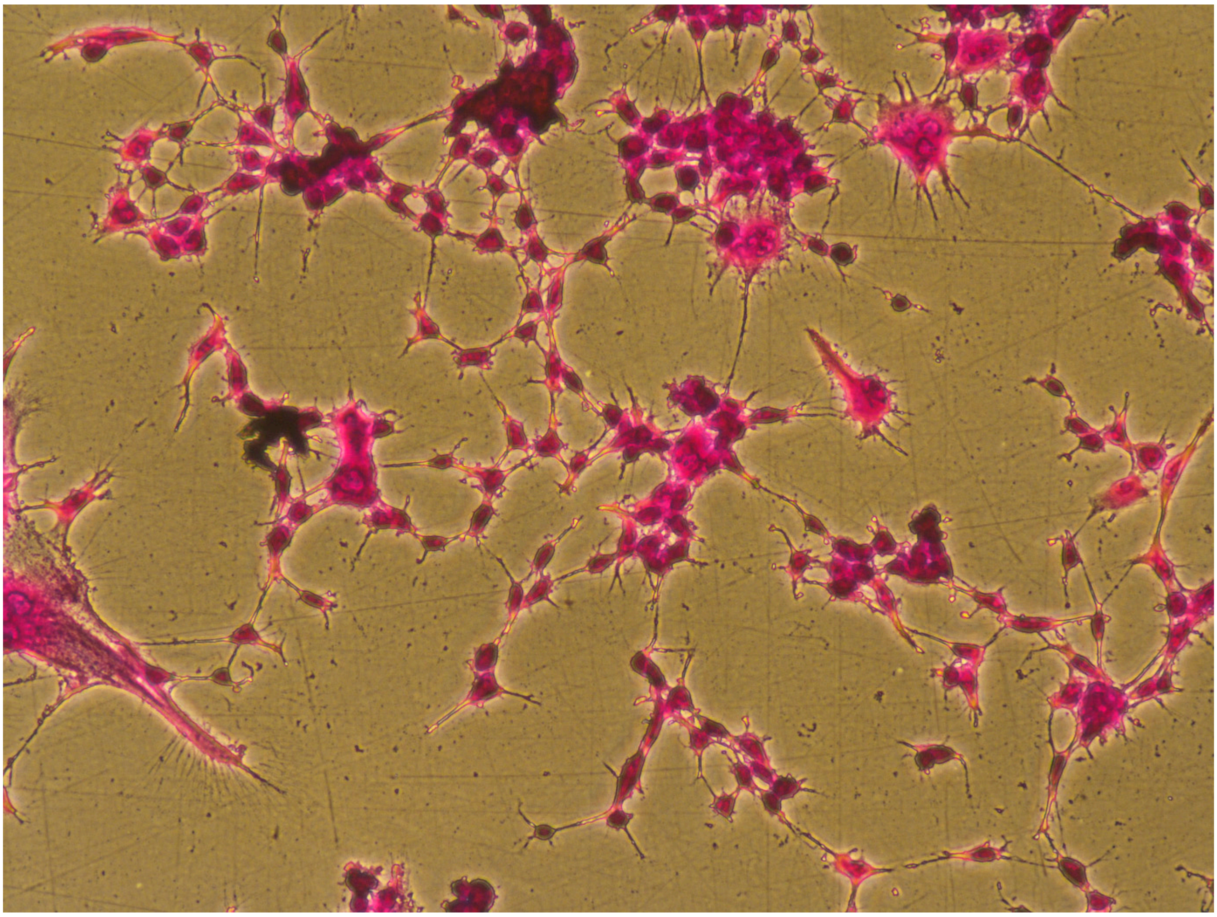

U87shp53 0nM AZD1152

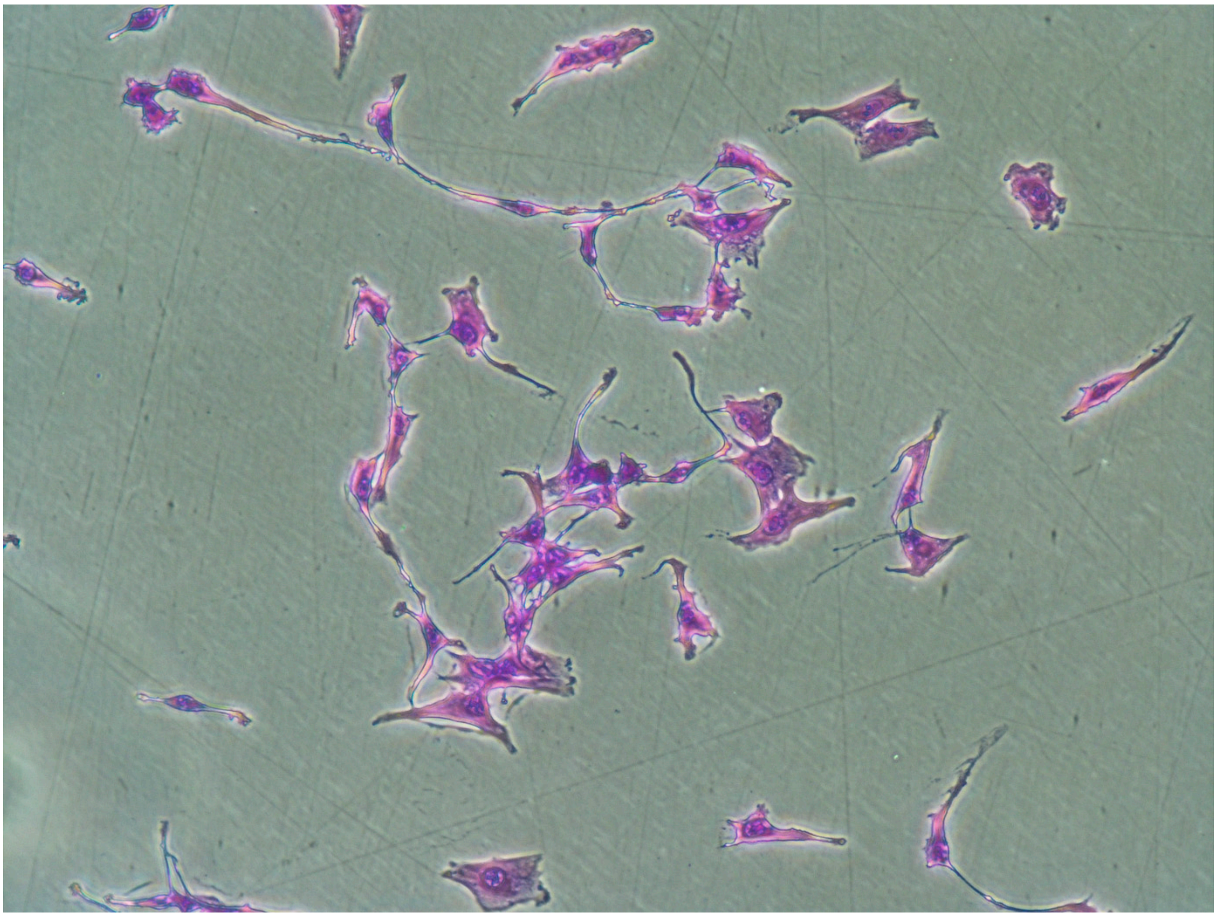

U87shp53 0nM AZD1152 + TTFields

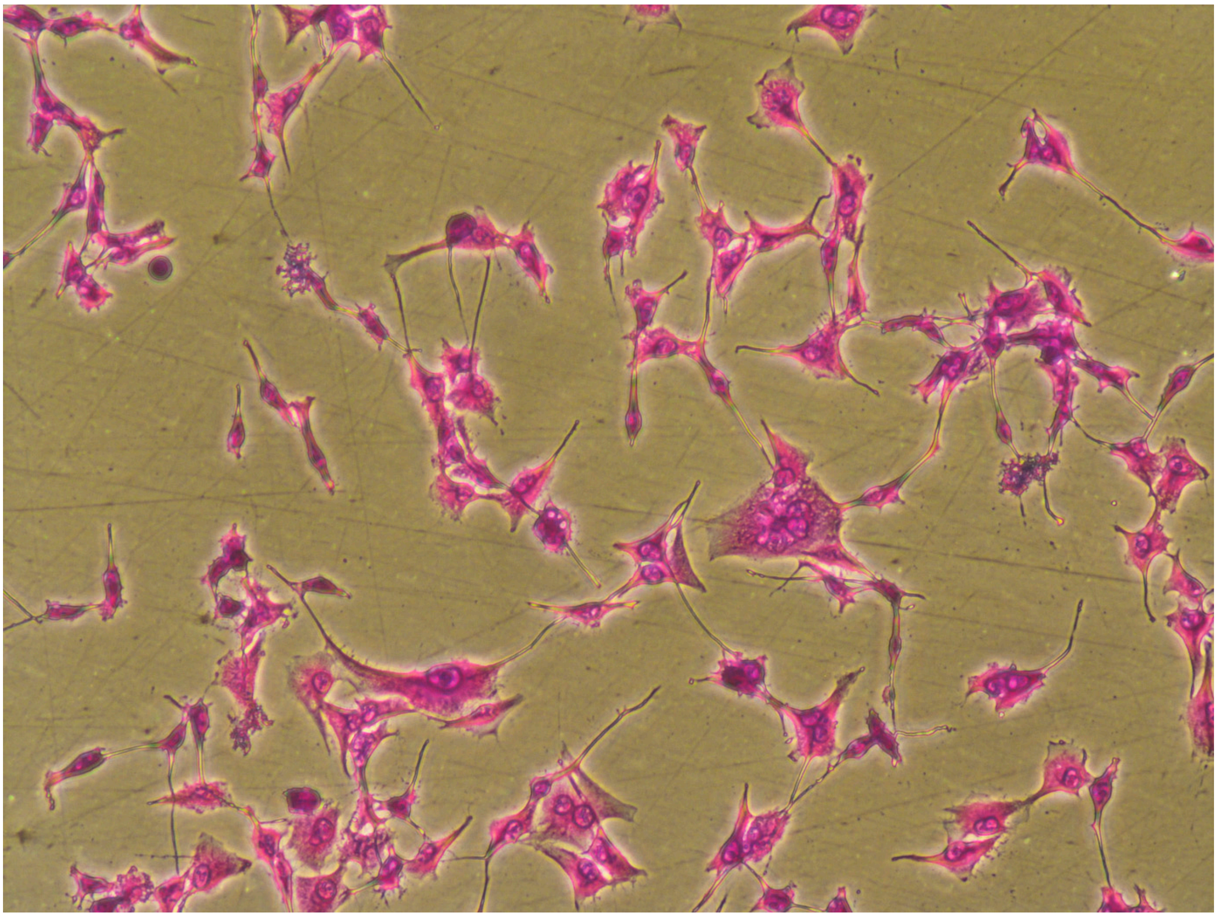

U87shp53 25 nM AZD1152

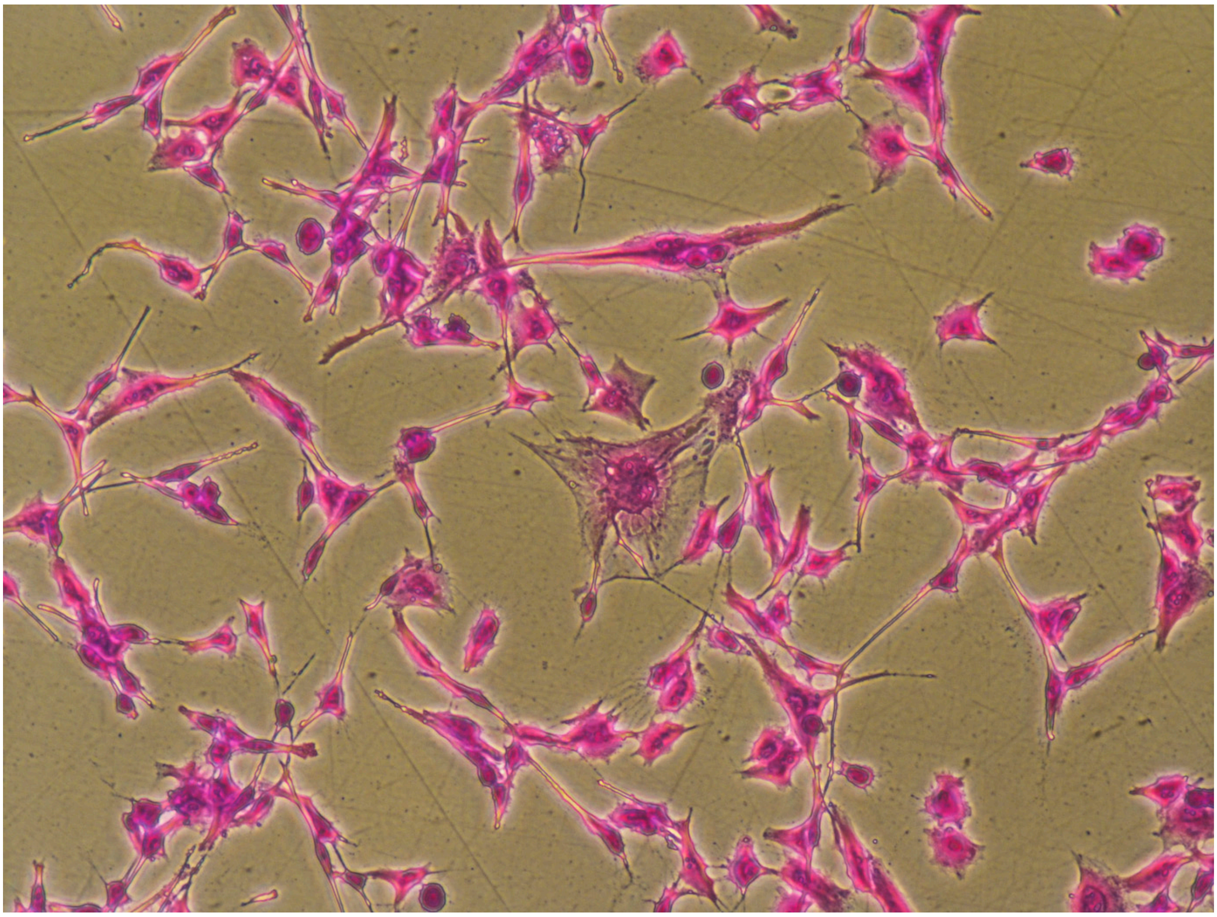

U87shp53 25 nM AZD1152 + TTFields

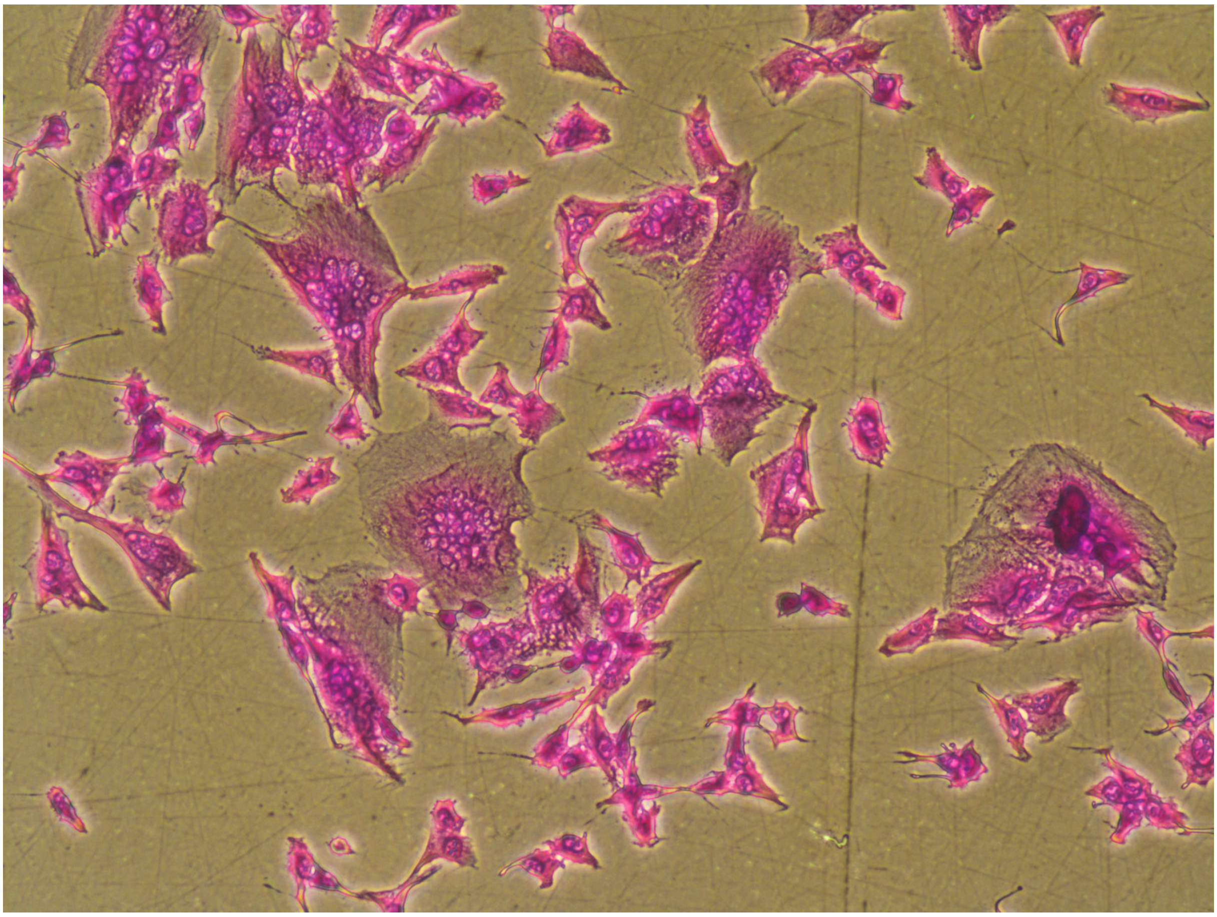

U87shp53 50 nM AZD1152

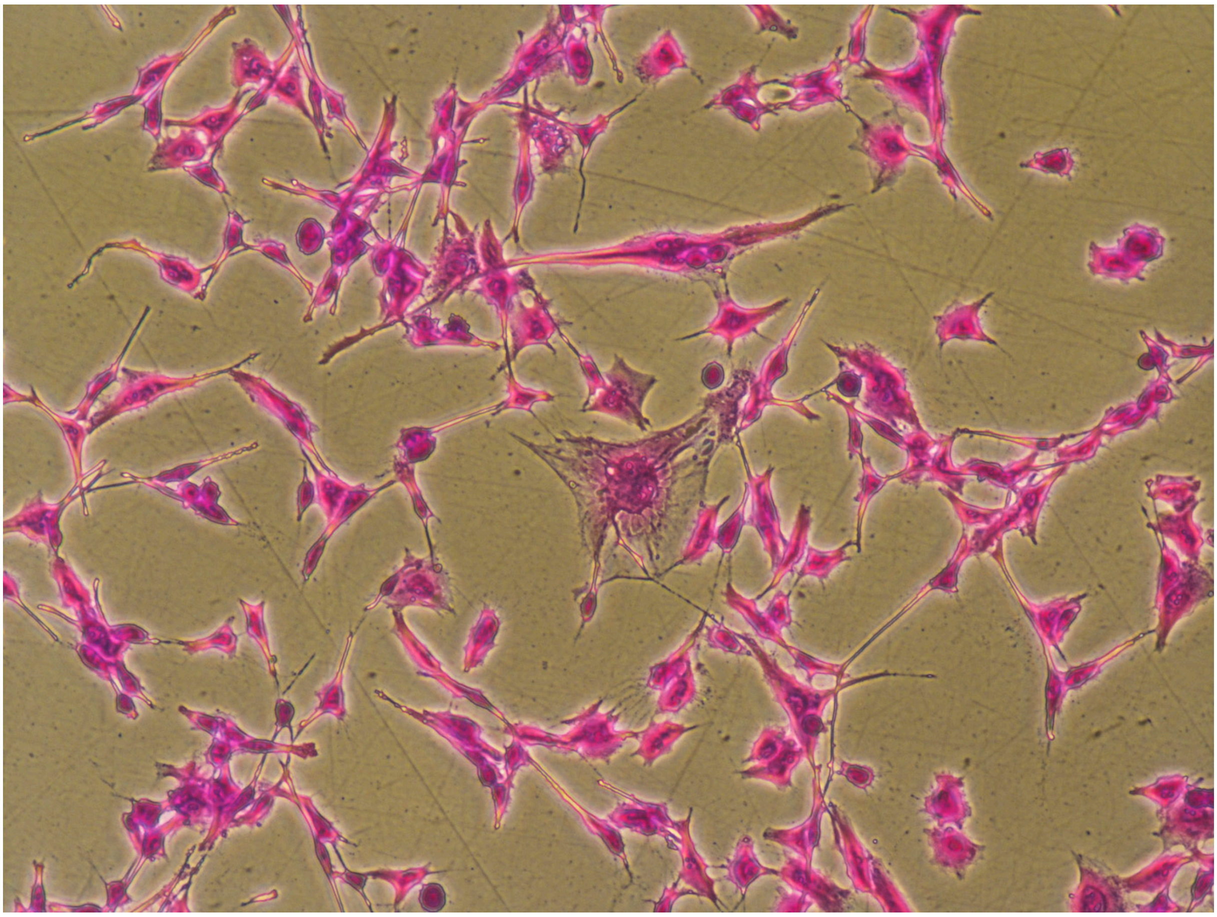

U87shp53 50 nM AZD1152 + TTFields

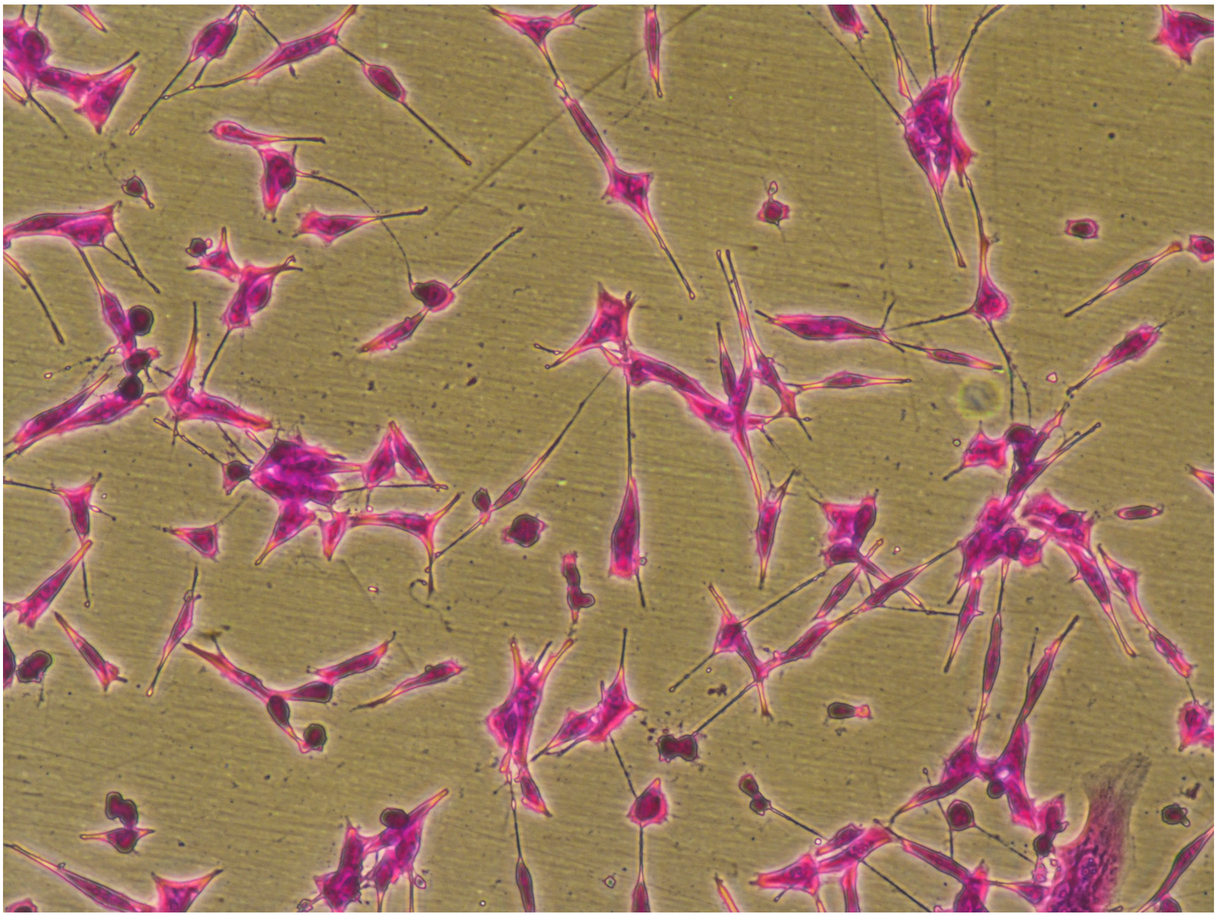

U87shp53 100 nM AZD1152

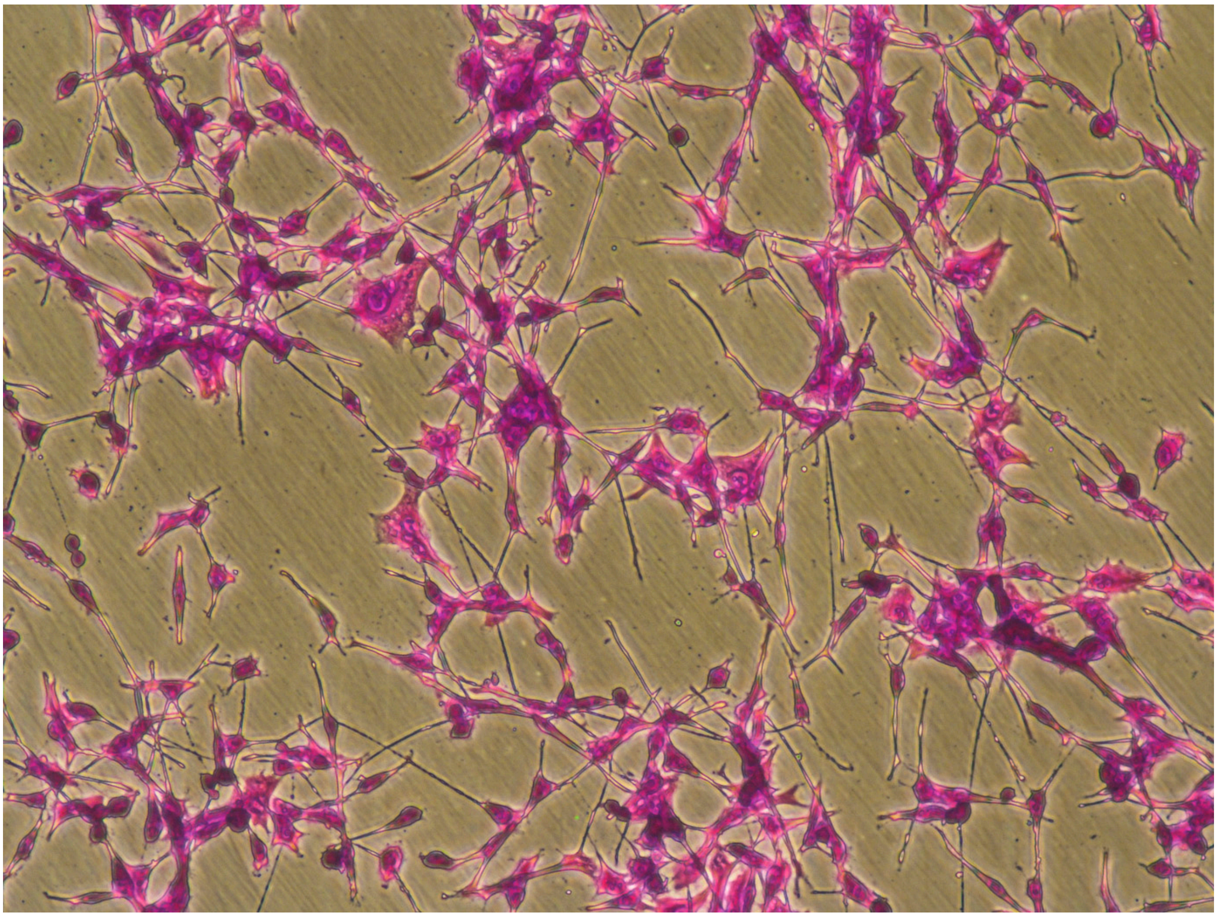

U87shp53 100 nM AZD1152 + TTFields

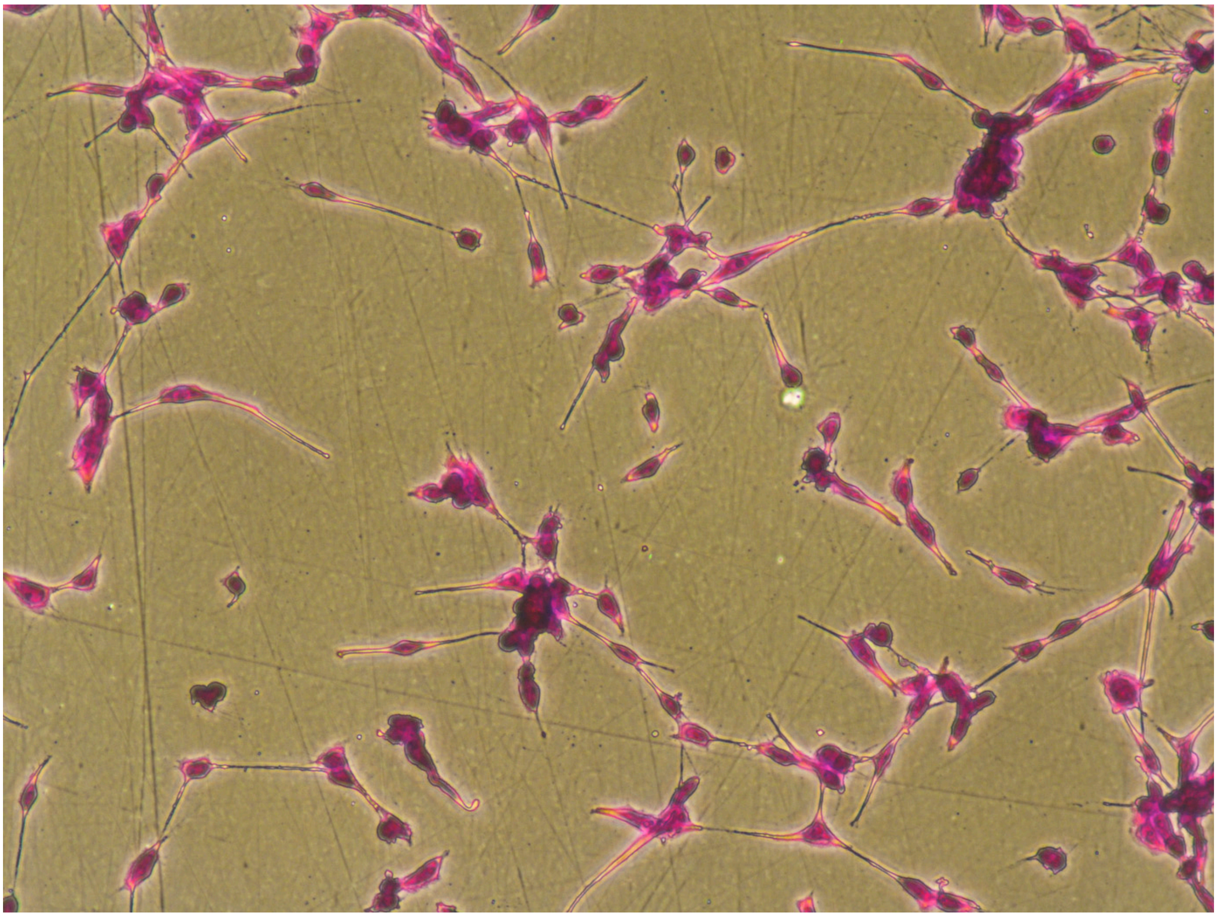

Figure S2: Enlarged (x10) views from Figure 1B showing cytotoxic effect in established cell lines. Formation of multinuclear and pyknotic cells was detected under inverted microscope after staining with crystal violet.

Figure S3

Percentage of multinuclear cells

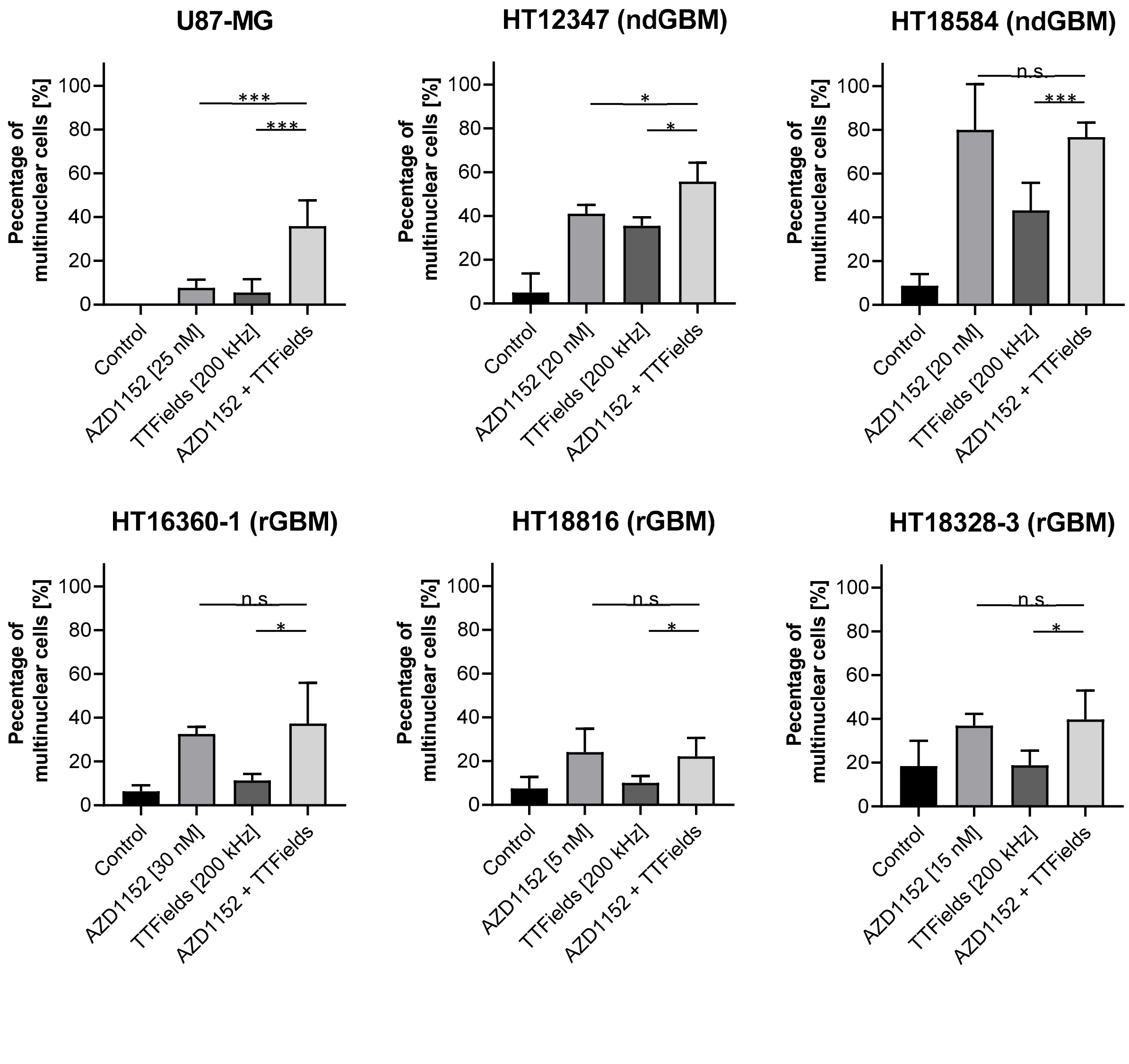

Figure S3. Percentage of multinuclear cells. Treatment with AZD1152 plus TTFields increase percentage of multinuclear cells in U87-MG and the primary glioblastoma cell cultures. Quantification of multinuclear cells after 72 h of treatment with AZD1152 alone, TTFields alone, and AZD1152 plus TTFields is shown. Data represent the mean value of nuclear cells (N ≥ 4). * p<0.05, ** p<0.01, *** p<0.001.

Figure S4

Enlarged views of morphological changes

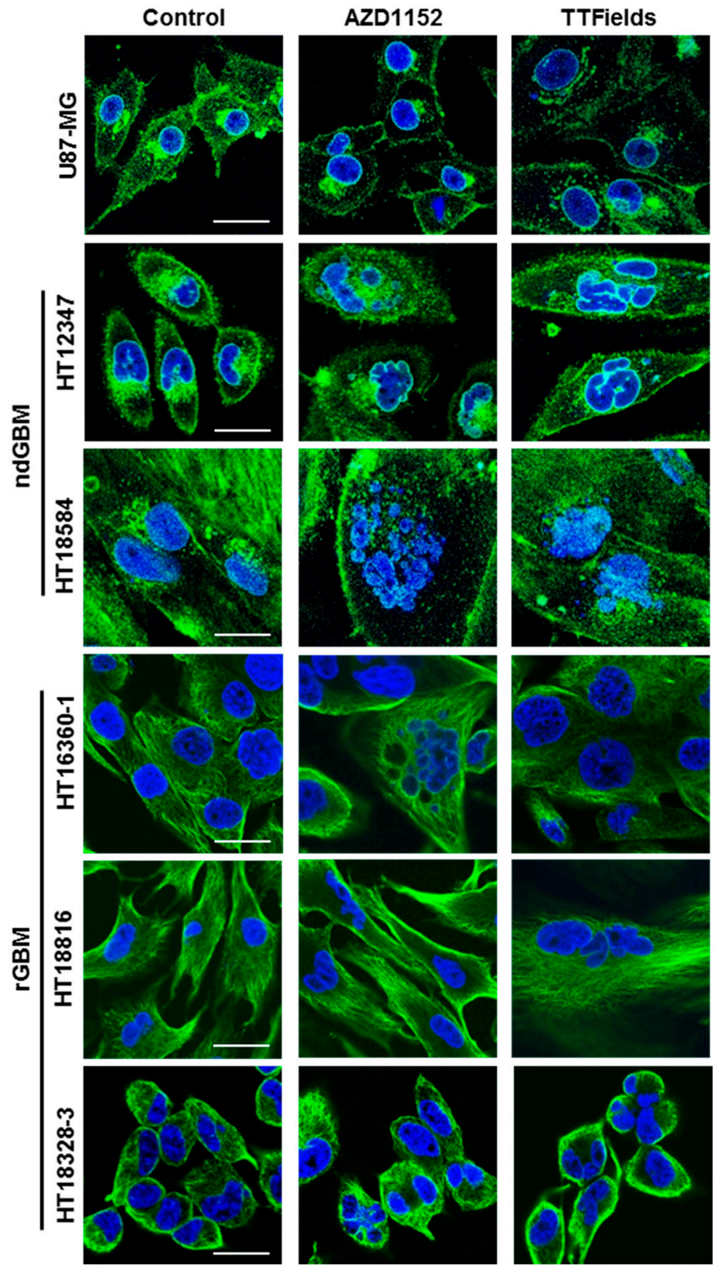

Figure S4: Morphological changes. Concomitant treatment of AZD1152 and TTFields enhances the morphological changes of the nucleus and cell shape in U87-MG and the primary glioblastoma cell cultures. Representative enlarged confocal laser scanning microscopic images after 72 h treatment with AZD1152 alone and TTFields alone are shown. Cell morphology was visualized in green with WGA (cell membrane staining, U87-MG and ndGBM) or anti-α-tubulin (cytoskeleton staining, rGBMs). DNA was stained with Hoechst 33342 (blue). The size scale in the control group corresponds to 25 µm.
